# Atrial Flow Regulator: Bridging Pediatric Patients With Pulmonary Arterial Hypertension to Lung Transplantation

**DOI:** 10.1016/j.jaccas.2025.106118

**Published:** 2025-11-17

**Authors:** Julia Moosmann, Anastasia Schleiger, Felix Berger, Peter Kramer

**Affiliations:** a Department of Congenital Heart Disease–Pediatric Cardiology, Deutsches Herzzentrum der Charité (DHZC), Berlin, Germany; b Charité–Universitätsmedizin Berlin, Corporate Member of Freie Universität Berlin and Humboldt-Universität zu Berlin, Berlin, Germany; c German Center for Cardiovascular Research (DZHK), Berlin, Germany

**Keywords:** atrial flow regulator, lung transplantation, pediatric, pulmonary arterial hypertension

## Abstract

**Background:**

Pulmonary arterial hypertension (PAH) in pediatric patients remains challenging, particularly in symptomatic patients with right heart failure despite optimal medical therapy. The use of an atrial flow regulator (AFR) to decompress the right ventricle has offered a new therapeutic approach for those patients and allows bridging for lung transplantation (LTX). Data on pretransplant decision-making, perioperative considerations, and post-transplant management in pediatric patients undergoing LTX are limited.

**Case Presentation:**

We report 2 pediatric patients with severe symptomatic PAH and right heart failure in whom an AFR was implanted before LTX. In both patients, AFR implantation resulted in clinical stabilization, allowing for successful listing and subsequent LTX. After LTX, both patients showed complete cardiac remodeling, and the AFR fenestration was closed percutaneously to minimize long-term complications.

**Conclusions:**

AFR implantation is a valuable bridging strategy to LTX in pediatric patients with therapy-refractory PAH, enabling clinical stabilization and safer perioperative management. Post-transplant transcatheter closure is reasonable to reduce long-term risks associated with persistent atrial shunting.

## Graphical Abstract

**Figure:**
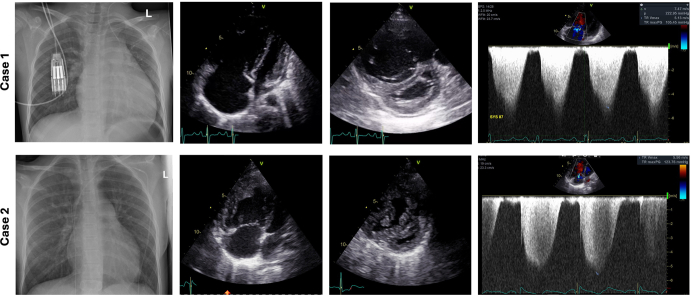

The atrial flow regulator (AFR, Occlutech) is a self-expandable percutaneously delivered double-disk device with a central fenestration, designed to create an interatrial shunt. This device was initially approved for adult patients with heart failure and reduced ejection fraction. In the pediatric population, its use has been successfully described in patients with congenital heart disease, including failing Fontan patients to create a right-to-left shunt, or in patients with cardiomyopathy and in patients with therapy-resistant pulmonary arterial hypertension (PAH).^1^^,^^2^ Clinical improvement or stabilization can be achieved after AFR implantation in patients with PAH by decompressing the right ventricle (RV), improving cardiac output and therefore end-organ function.^3^^,^^4^ In patients with RV failure refractory to optimal medical therapy, implantation of an AFR may serve as a feasible bridging strategy to lung transplantation (LTX).^5^ This report presents 2 pediatric cases of AFR implantation before LTX, focusing on pretransplant management, perioperative considerations, and post-transplant outcomes.

**Take-Home Message**

• In patients with severe PAH, atrial flow regulator implantation improves clinical symptoms and optimizes transplant candidacy, allowing for a safer bridge to lung transplantation.

## Case 1

A 9-year-old girl was diagnosed with PAH, prompted by the patient's reports of new-onset chest pain during exercise and multiple syncopal episodes during exertion.

The patient had a medical history of symptomatic focal epilepsy, diagnosed in early infancy, and showed mild facial dysmorphism. Previous genetic testing identified a variant in the *KCNQ2* gene (c.2068G>A; p.Val690Met), which is associated with early infantile epileptic encephalopathy and is not known to cause PAH. Under antiepileptic therapy, the patient had remained free of seizures and had developed appropriately for her age.

**Figure 1:**
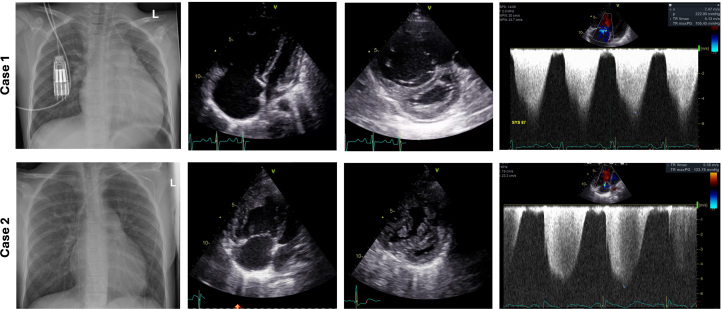
Imaging Findings in Patients With Pulmonary Arterial Hypertension Radiographic and echocardiographic imaging for (Top Row) case 1 and (Bottom Row) case 2. Both patients showed cardiomegaly and prominent PA segment, RA and RV dilatation, septal flattening, and suprasystemic RV pressure. PA = pulmonary artery; RA = right atrial; RV = right ventricular.

After initial stabilization and confirmation of pulmonary vasoreactivity, the patient was initiated on bosentan, sildenafil, and selexipag. Despite medical therapy, her clinical symptoms persisted, and she required readmission because of fulminant right heart failure in the setting of suprasystemic RV pressure. Echocardiography revealed a structurally normal heart with severe RV failure. The right atrium (RA) and RV were severely dilated. There was moderate tricuspid regurgitation (TR), septal flattening, and suprasystemic RV pressure (Figure 1).

**Figure 2:**
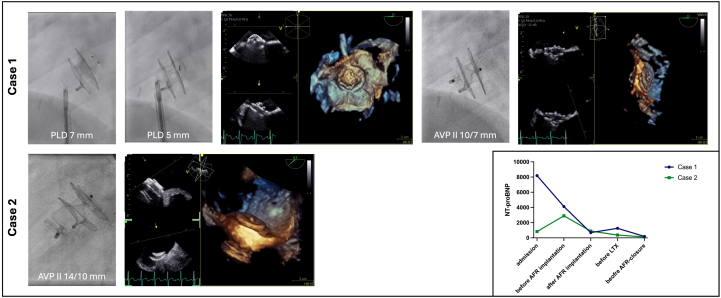
Closure of the Atrial Flow Regulator Fluoroscopic images and simultaneous 2D and 3D transesophageal echocardiography demonstrate (Top Row) attempted closure with paravalvular leak devices and final closure with the AVPII in case 1 and (Bottom Row) closure with the AVPII in case 2. (Bottom Right) Graph showing NT-proBNP levels at different time points: admission, before and after AFR implantation, before lung transplantation, and before AFR closure. 2D = two-dimensional; 3D = three-dimensional; AFR = atrial flow regulator; AVPII = Amplatzer vascular plug II; LTX = lung transplantation; NT-proBNP = N-terminal pro–B-type natriuretic peptide; PLD = paravalvular leak device.

Clinically, the patient was in reduced general condition and required admission to the pediatric intensive care unit for central line insertion under inotropic support and standby extracorporeal membrane oxygenation (ECMO). The medical treatment was changed to macitentan, sildenafil, and intravenous epoprostenol. Two days after admission, trans-septal puncture followed by implantation of an 8-mm AFR device was performed to decompress the right heart structures. The patient subsequently stabilized and was transferred to the general ward. No further syncopal events occurred, and N-terminal pro–B-type natriuretic peptide (NT-proBNP) levels normalized after AFR implantation (see graph in Figure 2).

Owing to an increased gradient across the AFR device as detected on transthoracic echocardiography, the patient underwent an additional cardiac catheterization 1 month after initial implantation. The gradient was successfully reduced by balloon dilatation using an ultra-high-pressure balloon (Conquest).

The patient was subsequently listed for LTX and successfully underwent bilateral LTX 1 month after admission. After LTX and remodeling of the RV, she showed normal mean pulmonary artery and RV pressure and an isolated left-to-right shunt across the AFR. The patient is currently 26 months post-LTX in good condition.

## Case 2

A 12-year-old girl presented to the emergency department with an upper airway infection and progressive decline in exercise capacity. She was a refugee and was diagnosed with PAH abroad during an episode of pneumonia 6 months before. She was started on oral pulmonary vasodilator treatment with sildenafil, macitentan, selexipag, and iloprost inhalation. She had experienced worsening of her symptoms in the previous month, including several syncopal events.

Echocardiography revealed a marked dilatation of the RV and RA, with suprasystemic RV pressures and moderate reduced RV systolic function. Moderate TR, interventricular septal flattening, and severe pulmonary artery dilatation was also shown (Figure 1). Genetic testing revealed a rare missense variant c.212C>A(p.Pro71Gln), which is regarded as likely pathogenic in the context of PAH.

The patient was admitted and initiated on intravenous epoprostenol therapy. After successful stabilization on epoprostenol, a permanent pump system was implanted 1 month after initial presentation. However, she showed insufficient clinical improvement and progressively deteriorated, presenting with recurrent chest pain, elevated NT-proBNP levels (see graph in Figure 2), and sinus tachycardia. Given the lack of response, the decision was made to create an atrial communication and implant an 8-mm AFR. After successful AFR implantation, the patient stabilized and was listed for LTX. NT-proBNP levels dropped within days after AFR implantation and normalized in the following months. No further syncopal events occurred.

After a 9 months on the transplant list, the patient underwent bilateral LTX, complicated by pneumonia and oxygenation issues requiring postoperative ECMO. Six months after LTX, the patient developed post-transplant lymphoproliferative disorder with B-cell lymphoma, which was successfully treated with rituximab. The patient is currently 14 months post-LTX in clinically stable condition.

## Interventional Aspects of the AFR

### AFR implantation

Case 1 underwent AFR implantation in our hybrid suite with ECMO on standby, whereas case 2 was treated in our standard catheterization laboratory. This decision was based on the individual clinical stability of each patient. Both procedures were performed under transesophageal echocardiography guidance and fluoroscopy. Trans-septal puncture was achieved using radiofrequency ablation, followed by ballon dilatation of the atrial septum with 8- and 10-mm Conquest balloons to create an atrial communication. Both patients immediately showed bidirectional shunting across the defect. Subsequently, an AFR device (5 and 8 mm) was successfully implanted in both patients without complications. Both patients showed a decrease in their oxygen saturation after implantation (Table 1). The AFR device was well seated in the interatrial septum, without obstructing surrounding structures (see, eg, case 1 in Video 1).

**Table 1:** Overview of Clinical and Interventional Parameters Associated With Perioperative Management

	Case 1	Case 2
Age (y)	9	12
Weight (kg)	31	33
Genetic finding	*KCNQ2* gene (c.2068G>A; p.Val690Met)	*SOX17* gene c.212C>A (p.Pro71Gln)
Waiting time to LTX (mo)	1	9
Medical therapy	Sildenafil, macitentan, epoprostenol i.v., nasal oxygen (2 L/min), milrinone	Sildenafil, macitentan, epoprostenol i.v. (pump), nasal oxygen (2 L/min)
AFR implantation after hospital admission (d)	17	6
Fluoroscopy time (min)	3.6	5
AFR size	8/5	8/5
Hemodynamics at AFR implantation (mm Hg)		
RA pressure	17	5
LA pressure	14	—
RA pressure with AFR	14	3
Gradient across AFR on TEE (mm Hg)	2	3
Saturation before AFR implantation (%)	97-99	96-98
Saturation after AFR implantation (%)	88-93	91-93
Reinterventions before LTX	Balloon dilatation of AFR (Conquest balloon)	Diagnostic cardiac catheterization
Days in ICU after LTX (d)	8	41
Total hospital stay after LTX (d)	76	78
Complications	Postoperative bleeding requiring rethoracotomy	Postoperative ECMO (4 d), tracheostomy (28 d), sepsis, PTLD 6 mo after LTX
AFR closure after LTX (mo)	4	10
Device used for AFR closure	AVPII 10 × 7 mm	AVPII 14 × 10 mm
Fluoroscopy time (min)	16	7.4
Hemodynamics at closure		
RA pressure	4	6
LA pressure	6	12
RVSP	30	—
Mean PA pressure	18	—
Wedge pressure	7	—
LVEDP	5	13
LVSP	140	110

AFR = atrial flow regulator; AVPII = Amplatzer vascular plug II; ECMO = extracorporeal membrane oxygenation; ICU = intensive care unit; i.v. = intravenous; LA = left atrium; LTX = lung transplantation; LVEDP = left ventricular end-diastolic pressure; LVSP = left ventricular systolic pressure; PA = pulmonary artery; PTLD = post-transplant lymphoproliferative disorder; RA = right atrium; RVSP = right ventricular systolic pressure; TEE = transesophageal echocardiography.

After the procedure, case 1 continued on intravenous heparin given the need for a permanent intravenous line for intravenous epoprostenol administration, and case 2 was transitioned to oral antiplatelet therapy with aspirin.

### AFR closure

Because of the presence of an interatrial shunt, antiplatelet therapy with aspirin was continued after LTX in both patients. However, considering the anticipated need for follow-up bronchoscopies and risk of associated bleeding, as well as the long-term risk for paradoxical embolism, the decision was made to close the AFR in both patients after LTX. Both patients showed marked cardiac remodeling, with normal RV size and function, trivial TR, normal RV pressure, and an isolated left-to-right shunt across the AFR.

In case 1, closure was performed 4 months after LTX. Initial attempts to close the device with the rectangular paravalvular leak device (Occlutech) at both 7 and 5 mm were not successful. The paravalvular leak devices were recommended because of their shape and their supposed ability to perfectly exclude the eccentric ball-connector of the AFR. However, both device configurations were too bulky, particularly the left atrial disk, and they were not released owing to concerns of left-sided thrombus formation (Figure 2). Eventually, the fenestration was closed with a 10-mm Amplatzer vascular plug II (AVPII; Abbott), showing a flat configuration in the left atrium and no residual shunt across the fenestration (Figure 2).

In case 2, the AFR was closed 10 months after LTX. The delayed closure compared with case 1 was attributed to her significant complications after transplantation. The atrial fenestration was closed with a 14-mm AVPII, which conformed nicely in the left atrium but appeared slightly bulkier within the RA owing to the its length (Figure 2). In both patients, the AVPII demonstrated a flawless, left-sided flat configuration at the site of the AFR, eliminating any residual shunting. The AVPII 10 × 7 mm size configured particularly well to achieve full closure, while the larger device was slightly bulkier on the RA side. Both procedures were performed under conscious sedation, with three-dimensional transesophageal echocardiography guidance and fluoroscopy. Antiplatelet therapy with aspirin was continued for another 6 months to ensure complete endothelialization of the device.

## Discussion

The AFR device has been successfully used in pediatric patients with PAH.^1^^,^^3^ Our cases demonstrate the effective use of AFR as a bridging strategy to LTX and the closure of the AFR after cardiac remodeling post-LTX.

In both of our patients, AFR implantation resulted in significant clinical improvement and most likely contributed to improved transplant candidacy. This was reflected by no further syncopal events and normalized NT-proBNP (Figure 2, graph). No device-related complications occurred during LTX. We hypothesize that the AFR implantation may also facilitate RV and left ventricular remodeling in the postoperative period. In these patients, the left ventricle is often chronically “deconditioned,” exhibiting diastolic dysfunction as a consequence of longstanding low cardiac output secondary to high pulmonary vascular resistance and reduced preload.^6^ The AFR might be beneficial in the postoperative phase to unload the left ventricle and enable a more gradual adaption to the increased preload during postoperative recovery.

In contrast to adult patients, where a residual atrial communication post-LTX may be of limited clinical relevance, it may warrant closure in pediatric patients, who have a comparably longer life expectancy. Therefore, closure of the AFR after successful LTX seems reasonable in this patient population. Furthermore, anticoagulation can typically be discontinued at 6 months after device closure, potentially improving long-term medical compliance by reducing daily medication burden.

After LTX, we observed complete remodeling of ventricular function, pressures, and ventricular volumes. This normalization of hemodynamics resulted in shunt reversal to a left-to-right shunt. In cases where atrial fenestration is no longer necessary, closure can be effectively achieved using an AVPII.

## Conclusions

The use of AFR represents a valuable therapeutic option in pediatric patients with PAH and RV failure as a bridging strategy to LTX. The achieved clinical stabilization enables safer perioperative management and improved transplant candidacy. Our cases illustrate that the AFR device can be safely managed throughout the transplantation process and closed effectively using an AVPII postoperatively to minimize long-term risks of an atrial shunt.

## Funding Support and Author Disclosures

The authors have reported that they have no relationships relevant to the contents of this paper to disclose.

## Appendix

Video 1 Atrial Flow Regulator in Motion Three-dimensional transesophageal echocardiography image of implanted AFR with shunt across the fenestration.
